# Patient-Directed Discharge Among Hospitalized Persons With Opioid Use Disorder in the Fentanyl Era: A Scoping Review

**DOI:** 10.1016/j.ajmo.2026.100130

**Published:** 2026-02-28

**Authors:** William M. Garneau, Severyn Kushmeliuk, Dustin P. Kee, Kelly A. Gebo, Megan E. Buresh

**Affiliations:** a Department of Medicine, Division of Hospital Medicine, Johns Hopkins Hospital, Baltimore, MD; b Department of Medicine, Icahn School of Medicine at Mount Sinai, New York, NY; c Department of Medicine, Johns Hopkins Hospital, Baltimore, MD; d Milken Institute School of Public Health, Washington, DC; e Department of Medicine, Division of Addiction Medicine, Johns Hopkins Bayview Medical Center, Baltimore, MD

**Keywords:** Against medical advice, Hospital-based car, Opioid use disorder, Patient-directed discharge

## Abstract

**Background:**

Patient-directed discharge (PDD), also known as discharge against medical advice, is associated with poor outcomes, such as increased hospital readmission and mortality. PDD is 10-20 times more common among persons with opioid use disorder (OUD). This scoping review sought to describe the reported incidence in the fentanyl era and characterize relevant patient level, clinical care level and systems level risk factors that may contribute to this phenomenon.

**Methods:**

A comprehensive assessment of the literature was conducted across 4 electronic databases: PubMed, CINAHL, Cochrane Library and Embase. The search strategy was designed to identify articles reporting PDD incidence and OUD in the era of fentanyl and other highly-potent synthetic opioids.

**Results:**

PDD incidence varies significantly by population; many studies report PDD rate in 10%-20% range and the incidence rate appears to be increasing over time. Younger patients with co-occurring stimulant use and OUD carry the highest rates of PDD. Studies examining the association between medications for OUD (MOUD) and decreased PDD report mixed findings, which likely reflects the impact of variability in medication selection and timing of administration. Consultation with addiction consult service (ACS) was not consistently associated with decreased PDD.

**Conclusions:**

Overall, there is increased rate of PDD in fentanyl era. More work is needed to characterize the relationship between MOUD provision and ACS with incidence of PDD.

## Introduction

Patient-directed discharge (PDD), also known as discharge against medical advice or before-medically advised discharge, occurs when a hospitalization is terminated prior to the timing determined by the medical care team. This phenomenon occurs in approximately 1%-2% of hospitalized patients but is 10-20 times more common among hospitalized persons with opioid use disorder (OUD).^1^, ^2^, ^3^ This is particularly notable as almost one quarter of the 5.7 million persons with OUD are hospitalized each year in the United States.^4^ PDD is associated with increased mortality, readmission, and overall costs for health systems.^5^, ^6^, ^7^, ^8^, ^9^, ^10^ The phenomenon has been described among various subpopulations of persons with OUD (eg, persons with endocarditis,^11^, ^12^, ^13^, ^14^, ^15^, ^16^, ^17^, ^18^ persons who use injection drugs^19^, ^20^, ^21^, ^22^, ^23^, ^24^, ^25^) and a wide variety of inpatient settings including community and academic hospitals.^1^, ^2^, ^3^^,^^26^, ^27^, ^28^, ^29^, ^30^, ^31^, ^32^, ^33^, ^34^, ^35^, ^36^, ^37^, ^38^, ^39^, ^40^, ^41^, ^42^, ^43^, ^44^, ^45^, ^46^, ^47^, ^48^, ^49^, ^50^ Prior work has summarized this topic however it did not include patients using fentanyl.^51^ The purpose of this scoping review is to synthesize existing knowledge on risk factors for PDD in the era of fentanyl and other highly potent synthetic opioids (HPSO) in the United States and Canada.

## Methods

A comprehensive assessment of the literature was conducted across 4 electronic databases: PubMed, CINAHL, Cochrane Library and Embase. Working with a reference librarian, relevant concepts were replicated across databases using applicable MeSH terms and subject headers. The search strategy was designed to identify articles reporting PDD incidence and OUD in the era of HPSO. Articles in which PDD was quantitively reported, including as a primary or secondary outcome were eligible for inclusion. Search terms included “opioid-related disorders,” “methadone,” “buprenorphine,” “medication for opioid use disorder”, “leaving against medical advice,” “treatment refusal,” “refusal of care,” “patient-directed discharge” and were limited to results after January 1, 2015. The initial search was performed on August 8, 2025; the same search terms were used to identify newly published manuscripts on January 8, 2026. A complete list of search terms and Boolean operators utilized is available in the **Appendix**.

The review utilized a PICO-TS framework and focused on hospitalized adult patients with opioid use disorder, receiving hospital-based care, compared with treatment as usual, with the primary outcome of patient-directed discharge during a single hospitalization in an acute care setting. It was conducted using Covidence systematic review software.^52^ Articles were considered eligible for inclusion if they met the following criteria: (1) focused on PDD in the context of OUD among hospitalized adult patients, (2) included original research or case series >5 patients, (3) were published in peer-reviewed journals and (4) published in English. Conference abstracts were excluded. Articles whose study time interval included any hospitalizations from 2015 and onward were included (eg, if the range of time included any portion of the year 2015 it was eligible for inclusion). Only articles that identified patients with OUD or injection drug use were included. Articles that identified persons using the term “substance use disorder” where this could also include other substances use disorders without OUD were excluded. Studies of an intervention for hospitalized patients with OUD were included in the review; when a reference group and intervention group were described, an average PDD rate was reported that includes both groups proportional to the size of each group. Given the focus on acute care for patients with co-morbid medical conditions, studies that included populations in “detoxification” units or specialized psychiatry units for withdrawal were excluded.

PDD can be formally documented using ICD-10-CM billing codes (Z53.21 “Procedure and treatment not carried out due to patient leaving prior to being seen by health care provider”)^53^ but is more often documented in a discharge summary or discharge disposition. There was general agreement in the reviewed literature on the concept of patient-directed discharge (PDD), typically defined as any hospitalization with discharge disposition as “Against Medical Advice” or “Eloped;” however, the specific language differs by health system.

Titles and abstracts of all studies were screened for relevance based on the eligibility criteria by one author (WG). One author independently reviewed the full texts of eligible studies for inclusion and performed data abstraction (WG). The reference lists of all included articles was reviewed to identify additional relevant studies not captured in the database search and screened for inclusion according to the same eligibility criteria as the database-identified records by one author (WG). In the course of the reverse citation search, several articles were identified incidentally and screened for inclusion; these records were screened and assessed using the same eligibility criteria by one author (WG) and reported separately as “hand search.”

While formal risk of bias assessment was not performed, the quality and relevance of included studies were appraised by evaluating study design, sample size, and methodology. Manuscripts were grouped according to the study population: studies were deemed originating from a single health system if all hospitals were part of the same system at the time of the study; studies were additionally identified either as originating from community or academic centers; finally, samples were identified as either nationally representative or not. Findings were synthesized to provide an overview of existing evidence on PDD in hospitalized patients with OUD.

## Results

**Figure 1:**
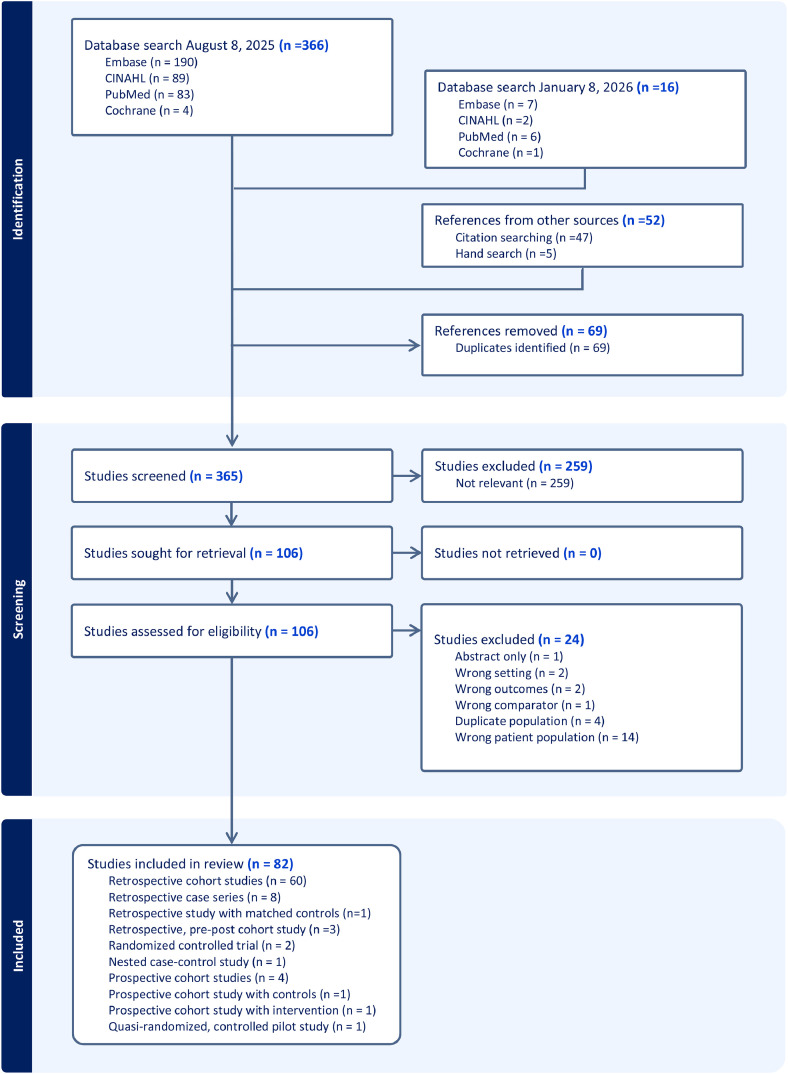
Preferred Reporting Items for Systematic Reviews and Meta-Analyses (PRISMA) flow diagram illustrating study selection for review.

A total of 366 manuscripts were identified using the original database search on August 8, 2025 and an additional 16 manuscripts were identified during follow up search on January 8, 2026; an additional 52 references were identified by reverse citation searching the bibliographies of eligible manuscripts (*n* = 47) and identified by hand search (*n* = 5). After duplicates (*n* = 69) and irrelevant studies were removed during title and abstract screening (*n* = 252) there were 106 studies included for full-text review. Of these, 24 studies were excluded (14 were excluded due to the wrong population [eg, only persons with alcohol use disorder], 4 included duplicate population as another manuscript, 2 did not include correct outcomes [eg, PDD], 2 were not in the right setting [eg, outpatient detoxification center], one was an abstract only, and one did not use a relevant comparator. A total of 82 studies were included in the review. Findings were organized at patient, clinical care and systems levels. A diagram illustrating the review process is displayed in Figure 1. Included studies are summarized in Table 1.

**Table 1:** Patient-directed Discharge Rates Among Hospitalized Patients With OUD

Author	Population	Study Design	n	Year	Location	Single Health System	Academic Center(s) Only	National Sample	PDD Rate
Alrawashdeh et al. (2023)^55^	Hospitalized patients with OUD included in National Inpatient Sample (USA)	Retrospective cohort study	1,27,158	2009-2015	USA	NO	NO	YES	5.6%
Appa et al. (2022)^5^	Hospitalized persons who use drugs in San Francisco, California	Retrospective cohort study	340	2013-2018	USA	NO	NO	NO	12.4%
Bhandari et al. (2022)^11^	Patients with IE in setting of drug use in West Virginia	Retrospective cohort study	553	2014-2018	USA	NO	YES	NO	19.9%
Bryant et al. (2025)^97^	Hospitalized patients in Florida	Retrospective case series	48	2021-2023	USA	YES	YES	NO	22.0%
Buehrle et al. (2017)^19^	Hospitalized patients with IDU in Pennsylvania	Retrospective cohort study	118	2013-2015	USA	YES	YES	NO	8.5%
Button et al. (2022)^98^	Patients hospitalized with OUD undergoing low dose buprenorphine induction in Oregon	Retrospective cohort study	72	2019-2020	USA	YES	YES	NO	2.8%
Casey et al. (2023)^78^	Patients with OUD treated with methadone hospitalized in Massachusetts	Retrospective cohort study	112	2016-2022	USA	YES	YES	NO	18.8%
Clifton et al. (2022)^108^	Hospitalized patients with OUD in Durham, North Carolina	Retrospective cohort study	512	2019-2021	USA	YES	YES	NO	7.0%
Compton et al. (2021)^3^	Adults with opioid-related conditions admitted to hospitals in Philadelphia, Pennsylvania	Retrospective cohort study	7,972	2017	USA	NO	NO	NO	15.6%
Coye et al. (2021)^62^	Hospitalized patients with infections who inject drugs in Florida	Retrospective cohort study	20,001	2016-2017	USA	NO	NO	NO	16.0%
Coyle et al. (2020)^8^	Adults with opioid-related infectious hospitalization in Michigan	Retrospective cohort study	23,164	2016-2018	USA	NO	NO	NO	9.9%
Eaton et al. (2020)^109^	Hospitalized patients with injection-drug use in Alabama	Retrospective cohort study	135	2015-2018	USA	YES	YES	NO	20.0%
Eaton et al. (2020)^30^	Hospitalized patients with injection-related infection in Alabama	Retrospective cohort study	83	2016-2017	USA	YES	YES	NO	14.4%
Englander et al. (2018)^125^	Hospitalized patients with drug-related infections long-term IV antibiotics in Oregon	Retrospective cohort study	45	2016	USA	YES	YES	NO	13.3%
Fanucchi et al. (2018)^121^	Hospitalized patients with drug-related infections in Kentucky	Prospective cohort study	42	2016	USA	YES	YES	NO	11.9%
Hazen et al. (2021)^9^	Hospitalized persons with injection drug use and OUD in Pennsylvania	Nested case-control study	1,264	2013-2018	USA	NO	NO	NO	15.6%
Horton et al. (2022)^59^	Hospitalized patients with opioid withdrawal in Mid-Atlantic region, USA	Retrospective cohort study	1,506	2017-2020	USA	YES	NO	NO	19.3%
Hrycko et al. (2023)^32^	Hospitalized persons who inject drugs in New York City, New York	Prospective cohort study	34	2020-2021	USA	YES	YES	NO	35.0%
Huang et al. (2018)^12^	Patients with IDU and repeat hospitalization for IE in North Carolina	Retrospective cohort study	22	2004-2017	USA	YES	YES	NO	9.1%
Iyer et al. (2024)^74^	Hospitalized pregnant patients with OUD in Philadelphia, Pennsylvania	Retrospective cohort study	194	2020-2022	USA	YES	YES	NO	27.3%
Jakubowski et al. (2024)^33^	Hospitalized persons with OUD in Bronx, NY	Retrospective study with matched controls	200	2019-2021	USA	YES	YES	NO	15.5%
Jakubowski et al. (2019)^110^	Hospitalized persons with OUD receiving overdose education in New York City, New York	Retrospective cohort study	50	2016-2017	USA	YES	YES	NO	18.0%
Jicha et al. (2019)^20^	Hospitalized persons with infection related to IDU in Kentucky	Retrospective cohort study	108	2012-2015	USA	YES	YES	NO	13.9%
Jo et al. (2021)^35^	Patients with OUD and endocarditis or osteomyelitis within 21 states in USA	Retrospective cohort study	1,407	2014-2018	USA	YES	NO	NO	18.3%
Karol et al. (2025)^82^	Hospitalized patients with OUD in Ohio	Quasi-randomized, controlled pilot study	214	2021-2022	USA	YES	YES	NO	9.3%
Kays et al. (2022)^36^	Patients with OUD at hospital in Kentucky	Retrospective cohort study	472	2018-2019	USA	YES	YES	NO	12.9%
Keegan et al. (2025)^21^	Hospitalized patients with OUD in Wisconsin	Retrospective, pre-post cohort study	227	2019-2021	USA	YES	YES	NO	37.9%
Kim et al. (2020)^13^	Hospitalizations for patients with OUD with serious infections in National Inpatient Sample (USA)	Retrospective cohort study	7,610	2016	USA	NO	NO	YES	19.1%
Kimmel et al. (2021)^54^	Hospitalizations for IDU-related IE in the National Inpatient Sample (USA)	Retrospective cohort study	7,259	2010-2015	USA	NO	NO	YES	14.2%
Klaire et al. (2023)^95^	Hospitalized patients with OUD treated with rapid methadone titration in Vancouver, Canada	Retrospective cohort study	98	2019-2020	Canada	YES	YES	NO	31.6%
Kociszewski et al. (2024)^56^	Hospitalizations for patients with IE and substance use in New Hampshire	Retrospective cohort study	144	2018-2020	USA	YES	NO	NO	38.2%
Levy et al. (2023)^65^	Hospitalized patients with OUD and amphetamine use in Alabama with IDU-related infections	Retrospective cohort study	370	2016-2021	USA	YES	YES	NO	23.4%
Lewis et al. (2022)^14^	Hospitalized patients with infection and OUD in Missouri	Prospective cohort study	166	2019-2021	USA	YES	NO	NO	63.3%
Liu et al. (2023)^38^	Hospitalized patients with OUD and stimulant use in King County, Washington	Retrospective cohort study	77	2019-2021	USA	YES	YES	NO	15.6%
Liu et al. (2024)^77^	Hospitalized patients with OUD treated with rapid methadone titration in Oregon	Retrospective cohort study	25	2022-2023	USA	YES	YES	NO	16.0%
Logan et al. (2025)^100^	Hospitalized patients with OUD in San Francisco treated with buprenorphine	Retrospective case series	37	2024-2025	USA	YES	YES	NO	10.8%
Marcovitz et al. (2024)^111^	Hospitalized patients with OUD in Tennessee	Randomized controlled trial	335	2019-2021	USA	YES	YES	NO	11.6%
Marks et al. (2019)^39^	Hospitalized patients with IDU and prolonged antimicrobial therapy in St Louis, Missouri	Retrospective cohort study	125	2016-2018	USA	YES	YES	NO	39.2%
Marks et al. (2020)^15^	Hospitalized patients who inject drugs with bacterial infection in St Louis, Missouri	Retrospective cohort study	293	2016-2019	USA	YES	YES	NO	51.2%
Marks et al. (2020)^71^	Hospitalized patients with infectious complications of OUD in St Louis, Missouri	Retrospective cohort study	220	2016-2019	USA	YES	YES	NO	38.6%
Martin et al. (2025)^76^	Hospitalized patients with OUD treated with rapid methadone titration in California	Retrospective case series	17	2023	USA	YES	YES	NO	5.6%
Meisner et al. (2020)^40^	Patients with IDU and IE in Pennsylvania	Retrospective cohort study	1,921	2013-2017	USA	NO	NO	NO	15.7%
Moreno et al. (2019)^10^	Hospitalized persons with OUD in Boston, Massachusetts	Retrospective cohort study	470	2011-2016	USA	YES	YES	NO	4.3%
Muller et al. (2023)^83^	Hospitalized persons on trauma service with OUD in Chicago, Illinois	Retrospective cohort study	113	2020-2021	USA	YES	YES	NO	6.2%
Nguyen et al. (2022)^112^	Hospitalized persons with OUD in Boston, Massachusetts	Prospective cohort study	40	2021	USA	YES	YES	NO	15.0%
Nolan et al. (2021)^64^	Adults hospitalized with IDU-related infection and OUD in St Louis, Missouri	Retrospective cohort study	262	2016-2019	USA	YES	YES	NO	40.8%
Novick et al. (2025)^22^	Hospitalized patients with OUD and wounds in Philadelphia	Retrospective cohort study	193	2023-2024	USA	YES	YES	NO	23.3%
Ovalle et al. (2020)^42^	Patients with IDU and upper extremity infection in Cincinnati, Ohio	Retrospective cohort study	396	2013-2015	USA	YES	YES	NO	15.9%
Paras et al. (2023)^84^	Patients hospitalized with IDU-related IE in Boston, Massachusetts	Retrospective, pre-post cohort study	46	2016-2020	USA	YES	YES	NO	8.7%
Price et al. (2020)^113^	Patients requiring IV antibiotics for IDU-related infections in Boston, Massachusetts	Retrospective cohort study	68	2018	USA	YES	YES	NO	14.6%
Priest et al. (2020)^49^	Patients with OUD hospitalized in Veterans Administration hospitals in United States	Retrospective cohort study	12,407	2017	USA	NO	NO	YES	5.7%
Racha et al. (2023)^46^	Patients with OUD treated with rapid methadone titration in Maryland	Retrospective case series	25	2018-2021	USA	YES	YES	NO	4.0%
Rapoport et al. (2021)^23^	Hospitalized patients with IDU-related infections in Boston, Massachusetts	Retrospective cohort study	448	2012-2015	USA	YES	YES	NO	12.0%
Ray et al. (2020)^85^	Hospitalized patients with IDU-related IE in Wisconsin	Retrospective, pre-post cohort study	107	2015-2016	USA	YES	NO	NO	12.1%
Rodger et al. (2018)^16^	Hospitalized patients with IE in London, Ontario	Retrospective cohort study	202	2015-2016	Canada	NO	NO	NO	16.8%
Rodger et al. (2023)^75^	Hospitalized pregnant patients with OUD in Toronto, Ontario	Retrospective case series	12	2016-2020	Canada	YES	YES	NO	0.0%
Rudasill et al. (2019)^87^	Hospitalized patients with IDU-related infections using National Readmissions Database (USA)	Retrospective cohort study	27,432	2010-2015	USA	NO	NO	YES	4.8%
Santos et al. (2021)^72^	Hospitalizations related to OUD in Pennsylvania	Retrospective cohort study	1,152	2016	USA	YES	YES	NO	6.4%
Schranz et al. (2019)^17^	Hospitalizations for patients with IDU-related endocarditis in North Carolina	Retrospective cohort study	2,602	2007-2017	USA	NO	NO	NO	13.0%
Schranz et al. (2023)^58^	Hospitalizations for patients with OUD associated IE in Nationwide Readmissions Database (USA)	Retrospective cohort study	3,222	2016	USA	NO	NO	YES	22.4%
Schuller et al. (2020)^69^	Hospitalized patients with IE and OUD in National Inpatient Sample (USA)	Retrospective cohort study	5760	2015	USA	NO	NO	YES	16.5%
Serota et al. (2021)^60^	Hospitalized persons with IDU in Florida	Retrospective cohort study	22,856	2016-2017	USA	NO	NO	NO	15.1%
Serota et al. (2023)^86^	Hospitalized patients with serious infections and IDU in Florida	Prospective cohort study with controls	129	2020-2022	USA	YES	YES	NO	27.9%
Serota et al. (2019)^24^	Hospitalized patients with IDU-related bacteremia in Georgia	Retrospective cohort study	46	2012-2017	USA	YES	NO	NO	26.1%
Seval et al. (2025)^81^	Hospitalized patients with OUD and infections in Connecticut, South Carolina, and Pennsylvania	Randomized controlled trial	171	2020-2023	USA	NO	NO	NO	14.1%
Shearer et al. (2024)^63^	Patients with OUD in National Inpatient Sample (USA)	Retrospective cohort study	6,95,504	2016-2019	USA	NO	NO	YES	10.7%
Shearer et al. (2024)^66^	Hospitalized persons with OUD in Minnesota	Retrospective cohort study	7,679	2016-2023	USA	YES	YES	NO	10.2%
Shen et al. (2024)^61^	Patients with opioid overdose hospitalization in Pennsylvania	Retrospective cohort study	13446	2018-2021	USA	NO	NO	NO	12.8%
Shields et al. (2023)^67^	Hospitalized patients with OUD receiving harm reduction services in Kentucky	Prospective cohort study with intervention	904	2020-2022	USA	YES	YES	NO	23.2%
Sikka et al. (2021)^114^	Hospitalized patients with IDU in Oregon	Retrospective cohort study	50	2018-2019	USA	YES	YES	NO	12.0%
Sredl et al. (2020)^68^	Hospitalizations with IDU-related infections in North Carolina	Retrospective cohort study	2,830	2010-2018	USA	NO	NO	NO	19.6%
Steiner et al. (2025)^80^	Hospitalized patients with OUD in San Francisco, California	Retrospective case series	108	2023	USA	YES	YES	NO	25.0%
Suzuki et al. (2020)^70^	Hospitalized persons with IDU in Boston, Massachusetts	Retrospective cohort study	84	2016-2018	USA	YES	YES	NO	16.7%
Tang et al. (2020)^99^	Hospitalized patients with OUD in Ontario, Canada	Retrospective case series	14	2015-2016	Canada	YES	YES	NO	7.1%
Thakarar et al. (2019)^18^	Hospitalized patients with IE in Maine	Retrospective cohort study	42	2013-2016	USA	YES	YES	NO	12.0%
Thakrar et al. (2024)^50^	Hospitalized patients in Philadelphia	Retrospective cohort study	226	2020-2021	USA	YES	YES	NO	19.9%
Thakrar et al. (2023)^43^	Hospitalized patients with OUD and IDU-related infection in United States	Retrospective cohort study	2,93,361	2020	USA	NO	NO	YES	11.3%
Thakrar et al. (2023)^79^	Hospitalized patients with OUD treated with short acting opioid therapy in Pennsylvania	Retrospective case series	23	2021-2022	USA	YES	YES	NO	43.5%
Tierney et al. (2022)^57^	Hospitalized persons with OUD in San Francisco, California	Retrospective cohort study	1,143	2016-2018	USA	YES	YES	NO	15.0%
Tsybina et al. (2021)^25^	Hospitalizations due to IDU in Saskatchewan, Canada	Retrospective cohort study	149	2018	Canada	YES	YES	NO	23.0%
Wang et al. (2020)^28^	Hospitalized patients with IDU in New Hampshire	Retrospective cohort study	147	2018-2019	USA	YES	NO	NO	41.5%
Zavodnick et al. (2023)^73^	Hospitalized patients with IDU related infections in Philadelphia	Retrospective cohort study	1,821	2017-2018	USA	YES	YES	NO	14.2%

IE = infective endocarditis; OUD = opioid use disorder; IDU = injection drug use; USA = United States of America

The majority were retrospective cohort studies (60/82) or retrospective case series (8/82). There were 9 studies with prospective or pre-post designs, including 2 randomized controlled trials (RCTs). The majority were from single health systems (60/82) and academic health centers (54/82). Over 90% of studies took place in the United States (77/82) and a small number took place in Canada (5/82).

### Incidence of PDD

Among the studies included in this review, the incidence of PDD among persons with OUD ranged from 0% to 63.3%. Of the 82 studies included, the weighted average for PDD rate was 10.6% among a total of 1,300,440 patients. The majority of manuscripts (44/82) reported PDD incidence rates of 10%-20%, however, several authors (11/82) also report PDD rates in the 30%-60% range. The studies that characterized PDD over the course of hospitalization found 30%-50% of PDD occur within the first 3 days of hospitalization.^43^^,^^54^ One study that characterized PDD in hospitalized patients with OUD over time found that the rate has increased from 7.5% in 2016 to 11.3% in 2020^43^ while another author reported a 12% rise per year in PDD incidence among persons who inject drugs between 2010 and 2015.^54^

### Patient Level Factors

There were 26 studies identified in the review that analyzed individual patient-level factors in relationship to PDD among hospitalized patients with OUD. These studies found that younger age,^3^^,^^5^^,^^35^^,^^54^, ^55^, ^56^, ^57^, ^58^, ^59^, ^60^, ^61^ methamphetamine or stimulant use,^5^^,^^38^^,^^43^^,^^55^^,^^56^^,^^59^, ^60^, ^61^, ^62^, ^63^, ^64^, ^65^, ^66^, ^67^, ^68^ nicotine use,^3^^,^^42^ housing instability,^3^^,^^5^^,^^60^ Medicaid or uninsured status,^35^^,^^42^^,^^54^^,^^56^^,^^58^^,^^61^^,^^64^^,^^69^ and opioid cravings or withdrawal^30^^,^^59^ were associated with a higher incidence of PDD. The literature was more mixed regarding the impact of sex,^3^^,^^5^^,^^30^^,^^35^^,^^54^^,^^56^^,^^58^^,^^60^^,^^61^^,^^64^ race,^5^^,^^35^^,^^54^^,^^55^^,^^57^^,^^60^^,^^61^ alcohol use,^5^^,^^56^^,^^58^^,^^61^ and mental illness^30^^,^^42^^,^^55^^,^^57^^,^^58^^,^^60^^,^^70^ on the incidence of PDD in this population. Patients with higher medical comorbidity score^5^^,^^54^^,^^57^ and maintenance MOUD at admission^57^^,^^70^ experienced a lower incidence of PDD. These findings are summarized in Table 2.

**Table 2:** Patient Risk Factors

Author	Population	Study Design	n	Year	Younger age	Male	Race	Methamph- etamine/ Stimulant use	Mental illness	Alcohol Use	Smoking/ Nicotine use	Increased medical comorbidities	Housing Instability	Uninsured/ Medicaid	Maintenance MOUD	Opioid Cravings/ Withdrawal	Wounds	Confirmed fentanyl use
Alrawashdeh et al. (2023)^55^	Hospitalized patients with OUD included in National Inpatient Sample (USA)	Retrospective cohort study	1,27,158	2009-2015	Increased Incidence	—-	Increased Incidence in non-White	Increased Incidence	Decreased Incidence	Increased Incidence	—-	—-	—-	—-	—-	—-	—-	—-
Appa et al. (2022)^5^	Hospitalized persons who use drugs in San Francisco, California	Retrospective cohort study	340	2013-2018	Increased Incidence	Similar Incidence	Similar Incidence	Increased Incidence	—-	Similar Incidence	—-	Decreased Incidence	Increased Incidence	—-	—-	—-	—-	—-
Compton et al. (2023)^3^	Adults with opioid-related conditions admitted to hospitals in Philadelphia, Pennsylvania	Retrospective cohort study	7,972	2017	Increased Incidence	Increased Incidence	—-	—-	—-	—-	Increased Incidence	—-	Increased Incidence	—-	—-	—-	—-	—-
Coye et al. (2021)^62^	Hospitalized patients with infections who inject drugs in Florida	Retrospective cohort study	20,001	2016-2017	—-	—-	—-	Increased Incidence									—-	—-
Eaton et al. (2020)^30^	Hospitalized patients with injection-related infection in Alabama	Retrospective cohort study	83	2016-2017	—-	Decreased Incidence	—-	—-	Similar Incidence	—-	—-	—-	—-	—-	—-	Increased Incidence	—-	—-
Horton et al. (2022)^59^	Hospitalized patients with opioid withdrawal in Delaware	Retrospective cohort study	1,506	2017-2020	Increased Incidence	—-	—-	Increased Incidence	—-	—-	—-	—-	—-	—-	—-	Increased Incidence	—-	—-
Jo et al. (2021)^35^	Patients with OUD and endocarditis or osteomyelitis within 21 states in USA	Retrospective cohort study	1,407	2014-2018	Increased Incidence	Similar Incidence	Similar Incidence	Similar Incidence	—-	—-	—-	—-	—-	Increased Incidence	—-	—-	—-	—-
Kimmel et al. (2021)^54^	Hospitalizations for IDU-related IE in the National Inpatient Sample (USA)	Retrospective cohort study	7,259	2010-2015	Increased Incidence	Decreased Incidence	Similar Incidence	—-	—-	—-	—-	Decreased Incidence	—-	Increased Incidence	—-	—-	—-	—-
Kociszewski et al. (2024)^56^	Hospitalizations for patients with IE and substance use in Northeast United States	Retrospective cohort study	144	2018-2020	Increased Incidence	Similar Incidence	—-	Increased Incidence	—-	Similar Incidence	—-	—-	—-	Increased Incidence	—-	—-	—-	—-
Levy et al. (2023)^65^	Hospitalized patients with OUD and amphetamine use in Alabama with IDU-related infections	Retrospective cohort study	370	2016-2021	—-	—-	—-	Increased Incidence	—-	—-	—-	—-	—-	—-	—-	—-	—-	—-
Liu et al. (2023)^38^	Hospitalized patients with OUD and stimulant use in King County, Washington	Retrospective cohort study	77	2019-2021	—-	—-	—-	Increased Incidence	—-	—-	—-	—-	—-	—-	—-	—-	—-	—-
Nolan et al. (2021)^64^	Adults hospitalized with IDU-related infection and OUD in St Louis, Missouri	Retrospective cohort study	262	2016-2019	—-	Decreased Incidence	—-	Methamph- etamine Increased Incidence, Cocaine Similar Incidence	—-	—-	—-	—-	Similar Incidence	Increased Incidence	—-	—-	—-	—-
Novick et al. (2025)^22^	Hospitalized patients with OUD and wounds in Philadelphia	Retrospective cohort study	193	2023-2024	—-	—-	—-	—-	—-	—-	—-	—-	—-	—-	—-	—-	Increased Incidence	—-
Ovalle et al. (2020)^42^	Patients with IDU and upper extremity infection in Cincinnati, Ohio	Retrospective cohort study	308	2013-2015	—-	—-	—-	—-	Increased Incidence	—-	Increased Incidence	—-	—-	Increased Incidence	—-	—-	—-	—-
Schuller et al. (2020)^69^	Hospitalized patients with IE and OUD in National Inpatient Sample (USA)	Retrospective cohort study	5760	2015	—-	—-	—-	—-	—-	—-	—-	—-	—-	Increased Incidence	—-	—-	—-	—-
Schranz et al. (2023)^58^	Hospitalizations for patients with OUD associated IE in Nationwide Readmissions Database (USA)	Retrospective cohort study	3,222	2016	Increased Incidence	Decreased Incidence	—-	—-	Similar Incidence	Similar Incidence	—-	—-	—-	Increased Incidence	—-	—-	—-	—-
Serota et al. (2021)^60^	Hospitalized persons with IDU in Florida	Retrospective cohort study	22,856	2016-2017	Increased Incidence	Decreased Incidence	Decreased Incidence in Black Patients	Increased Incidence	Decreased Incidence with Depression	—-	—-	—-	Increased Incidence	—-	—-	—-	—-	—-
Shearer et al. (2024)^63^	Patients with OUD in National Inpatient Sample (USA)	Retrospective cohort study	6,95,504	2016-2019	—-	—-	—-	Increased Incidence	—-	—-	—-	—-	—-	—-	—-	—-	—-	—-
Shearer et al. (2024)^66^	Hospitalized persons with OUD in Minnesota	Retrospective cohort study	7,679	2016-2023	—-	—-	—-	Increased Incidence	—-	—-	—-	—-	—-	—-	—-	—-	—-	—-
Shen et al. (2024)^61^	Patients with opioid overdose hospitalization in Pennsylvania	Retrospective cohort study	13446	2018-2021	Increased Incidence	Increased Incidence	Similar Incidence	Increased Incidence	—-	Similar Incidence	—-	—-	—-	Increased Incidence	—-	—-	—-	—-
Shields et al. (2023)^67^	Hospitalized patients with OUD receiving harm reduction services in Kentucky	Prospective cohort study with intervention	904	2020-2022	—-	—-	—-	Increased Incidence	—-	—-	—-	—-	—-	—-	—-	—-	—-	—-
Sredl et al. (2020)^68^	Hospitalizations with IDU-related infections in North Carolina	Retrospective cohort study	2830	2010-2018	—-	—-	—-	Increased Incidence	—-	—-	—-	—-	—-	—-	—-	—-	—-	—-
Suzuki et al. (2020)^70^	Hospitalized persons with IDU in Boston, Massachusetts	Retrospective cohort study	84	2016-2018	—-	—-	—-	—-	Increased Incidence for PTSD	—-	—-	—-	—-	—-	Decreased Incidence	—-	—-	—-
Thakrar et al. (2024)^50^	Hospitalized patients in Philadelphia	Retrospective cohort study	226	2020-2021	—-	—-	—-	—-	—-	—-	—-	—-	—-	—-	—-	—-	—-	Increased Incidence
Thakrar et al. (2023)^43^	Hospitalized patients with OUD and IDU-related infection in United States	Retrospective cohort study	2,93,361	2020	—-	—-	—-	Increased Incidence	—-	—-	—-	—-	—-	—-	—-	—-	—-	—-
Tierney et al. (2022)^57^	Hospitalized persons with OUD in San Francisco, California	Retrospective cohort study	1,143	2016-2018	Increased Incidence	—-	Increased Incidence in White Non-Hispanic	—-	Decreased Incidence with Anxiety	—-	—-	Decreased Incidence	—-	—-	Decreased Incidence	—-	—-	—-

IE = Infective endocarditis; OUD = opioid use disorder

### Clinical Care Factors

Among the studies identified in the scoping review, 23 analyzed how an aspect of clinical care (eg, provision of MOUD, ACS consultation) for hospitalized patients with OUD was associated with risk of PDD. Six studies found that MOUD was associated with a lower incidence of PDD^28^^,^^36^^,^^55^^,^^64^^,^^71^^,^^72^ however, other work did not find a difference^30^^,^^33^^,^^35^^,^^57^^,^^70^ and one study found a higher rate of PDD in patients that were provided MOUD (16.9% vs. 12.1%).^73^

Regarding specific MOUD treatment protocols, one study of rapid titration of methadone found a lower incidence of PDD in a cohort of pregnant participants with OUD.^74^ Low incidence of PDD was also reported in other studies with rapid methadone titration protocols but these studies did not include a comparator group.^46^^,^^75^, ^76^, ^77^, ^78^ Other inpatient treatment strategies include the use of short-acting opioid agonist treatment (sOAT) in addition to MOUD. One study of sOAT noted decreased incidence of PDD among patients who received higher doses of sOAT^79^ (44% vs. 69%); while another retrospective sOAT study reported patient outcomes (25% PDD) but did not provide a comparator group.^80^ One RCT found a higher rate of PDD among patients treated with injectable buprenorphine (16.7%) compared to treatment as usual (11.8%).^81^

Several studies have characterized outcomes among patients receiving addiction consult services (ACS). Authors have reported no change in the incidence of PDD^33^^,^^82^^,^^83^ with ACS although one manuscript did report a decreased rate (16% vs. 49%).^39^ Another treatment approach that has been tested is the use of multi-disciplinary teams including specialists from addiction medicine, infectious diseases, and other disciplines to provide inpatient care for patients with OUD. Authors have found that these teams were not associated with lower incidence of PDD.^84^, ^85^, ^86^ Lastly, in terms of clinical interventions and PDD; 2 studies noted that hospitalized patients with OUD who underwent surgical source control experienced a lower incidence of PDD.^5^^,^^54^ These findings are summarized in Table 3.

**Table 3:** Clinical Risk Factors

Author	Population	Study Design	n	Year	Rapid methadone titration	Short-acting opioid agonist	MOUD Treatment	Addiction Consult	Specialized Team	PICC line placement	Surgical source control	Use of long-acting injectable buprenorphine
Alrawashdeh et al. (2023)^55^	Hospitalized patients with OUD included in National Inpatient Sample (USA)	Retrospective cohort study	1,27,158	2009-2015	—-	—-	Decreased incidence	—-	—-	—-	—-	—-
Appa et al. (2022)^5^	Hospitalized persons who use drugs in San Francisco, California	Retrospective cohort study	340	2013-2018	—-	—-		—-	—-	Decreased incidence	Decreased incidence	—-
Eaton et al. (2020)^30^	Hospitalized patients with injection-related infection in Alabama	Retrospective cohort study	83	2016-2017	—-	—-	Similar incidence	—-	—-	—-	—-	—-
Iyer et al. (2024)^74^	Hospitalized pregnant patients with OUD in Philadelphia, Pennsylvania	Retrospective cohort study	194	2020-2022	Decreased incidence	—-	—-	—-	—-	—-	—-	—-
Jakubowski et al. (2024)^33^	Hospitalized persons with OUD in Bronx, NY	Retrospective study with matched controls	200	2019-2021	—-	—-	Similar incidence	Similar incidence	—-	—-	—-	—-
Jo et al. (2021)^35^	Patients with OUD and endocarditis or osteomyelitis within 21 states in USA	Retrospective cohort study	1,407	2014-2018	—-	—-	Similar incidence	—-	—-	—-	—-	—-
Karol et al. (2025)^82^	Hospitalized patients with OUD in Ohio	Quasi-randomized, controlled pilot study	214	2021-2022	—-	—-	—-	Similar incidence	—-	—-	—-	—-
Kays et al. (2022)^36^	Patients with OUD at hospital in Kentucky	Retrospective cohort study	472	2018-2019	—-	—-	Decreased incidence	—-	—-	—-	—-	—-
Kimmel et al. (2021)^54^	Hospitalizations for IDU-related IE in the National Inpatient Sample (USA)	Retrospective cohort study	7,259	2010-2015	—-	—-	—-	—-	—-	—-	Decreased incidence	—-
Marks et al. (2020)^71^	Hospitalized patients with infectious complications of OUD in St Louis, Missouri	Retrospective cohort study	220	2016-2019	—-	—-	Decreased incidence	—-	—-	—-	—-	—-
Marks et al. (2019)^39^	Hospitalized patients with IDU and prolonged antimicrobial therapy in St Louis, Missouri	Retrospective cohort study	125	2016-2018	—-	—-	—-	Decreased incidence	—-	—-	—-	—-
Muller et al. (2023)^83^	Hospitalized persons on trauma service with OUD in Chicago, Illinois	Retrospective cohort study	113	2020-2021	—-	—-	—-	Similar incidence	—-	—-	—-	—-
Nolan et al. (2021)^64^	Adults hospitalized with IDU-related infection and OUD in St Louis, Missouri	Retrospective cohort study	262	2016-2019	—-	—-	Decreased incidence	—-	—-	—-	—-	—-
Paras et al. (2023)^84^	Patients hospitalized with IDU-related IE in Boston, Massachusetts	Retrospective, pre-post cohort study	46	2016-2020	—-	—-	—-	—-	Similar incidence	—-	—-	—-
Ray et al. (2020)^85^	Hospitalized patients with IDU-related IE in Wisconsin	Retrospective, pre-post cohort study	107	2015-2016	—-	—-	—-	—-	Similar incidence	—-	—-	—-
Santos et al. (2021)^72^	Hospitalizations related to OUD in Pennsylvania	Retrospective cohort study	1,152	2016	—-	—-	Decreased incidence	—-	—-	—-	—-	—-
Serota et al. (2023)^86^	Hospitalized patients with serious infections and IDU in Florida	Prospective cohort study with controls	129	2020-2022	—-	—-	—-	—-	Decreased incidence	—-	—-	—-
Seval et al. (2025)^81^	Hospitalized patients with OUD and infection in Pennsylvania, South Carolina, Connecticut	Randomized controlled trial	171	2020-2023	—-	—-	—-	—-	—-	—-	—-	Increased incidence
Suzuki et al. (2020)^70^	Hospitalized persons with IDU in Boston, Massachusetts	Retrospective cohort study	84	2016-2018	—-	—-	Similar incidence	—-	—-	—-	—-	—-
Thakrar et al. (2023)^79^	Hospitalized patients with OUD treated with short acting opioid therapy in Pennsylvania	Retrospective case series	23	2021-2022	—-	Decreased incidence		—-	—-	—-	—-	—-
Tierney et al. (2022)^57^	Hospitalized persons with OUD in San Francisco, California	Retrospective cohort study	1,143	2016-2018	—-	—-	Similar incidence	—-	—-	—-	—-	—-
Wang et al. (2020)^28^	Hospitalized patients with IDU in New Hampshire	Retrospective cohort study	147	2018-2019	—-	—-	Decreased incidence	—-	—-	—-	—-	—-
Zavodnick et al. (2023)^73^	Hospitalized patients with IDU related infections in Philadelphia	Retrospective cohort study	1,821	2017-2018	—-	—-	Increased incidence	—-	—-	—-	—-	—-

IE = Infective endocarditis; OUD = opioid use disorder; PICC = peripherally-inserted central catheter; MOUD = medications for opioid use disorder.

### Systems-Level Factors

There were 2 manuscripts that reported on systems level factors associated with PDD in terms of hospitalized patients with OUD.^54^^,^^58^ One study that used the National Inpatient Sample found that there was a decreased odds of PDD in large hospitals as well as hospitals in the southern United States.^54^ Another author analyzed a nationwide readmissions database and found that patients hospitalized in metropolitan areas experienced higher rate of PDD.^58^ These findings are summarized in Table 4.

**Table 4:** Systems Level Factors

Author	Population	Study Design	n	Year	Metropolitan Area	Large Hospital	Hospital Location	Teaching Hospital
Kimmel et al. (2021)^54^	Hospitalizations for injection drug use related IE in the National Inpatient Sample in USA	Retrospective cohort study	7,259	2010-2015	—-	Decreased Risk	Decreased Risk in Southern USA	Similar Risk
Schranz et al. (2023)^58^	Hospitalizations for IE associated with OUD disorder in Nationwide Readmissions Database	Retrospective cohort study	3,222	2016	Increased Risk	—-	—-	—-

IE = Infective endocarditis; OUD = opioid use disorder.

## Discussion

This review is notable for several findings. First, PDD incidence varies significantly by specific population of persons with OUD; many studies report PDD rate in 10%-20% range and the incidence rate appear to be increasing over time. Second, younger patients with co-occurring stimulant use and OUD are at uniquely high risk of PDD. Third, MOUD appears to mitigate the incidence of PDD but dose, medication selection and timing are important. Lastly, consultation with ACS was not reliably associated with decreased PDD. However significant confounding by indication (eg, patients with more severe OUD receive ACS) is present in existing work and PDD is not the primary outcome assessed in the majority of ACS studies that do examine it.

### Incidence

The variation in PDD incidence is likely related to the diverse study populations, high level of heterogeneity between studies, and clinical scenarios included in this review. For instance, several manuscripts that include a clinical intervention report low rates of PDD (eg, Rodger et al. reported a PDD rate of 0% among 12 pregnant patients treated with high dose methadone titration and slow-release oral morphine in Canada.)^75^ This review also spans studies in which there was little clinical intervention for OUD; for instance Marks et al. describe clinical scenarios in which most patients do not receive MOUD and PDD rates approach 50%.^39^ The time frame for studies also likely contributes to variability in PDD rates, as noted by increasing rate of PDD.^43^ For example, some of the studies reporting low PDD rates (4%-5%) focused on hospitalizations from 2009 to 2015, an era that largely predate the appearance of HPSOs in the US drug supply^55^^,^^87^

### Patient Level Factors

All 11 studies that included age found that PDD occurred more frequently among patients who were younger. Most studies (8/11) treated age as a continuous variable; among studies that used categories of age, Kimmel et al. reports that age 18-24 years has the highest OR of PDD, Serota et al. reports age 18-34 has highest adjusted RR of PDD and Shen et al. reports age <44 had highest OR of PDD.^54^^,^^60^^,^^61^ Younger age may be associated with higher PDD rates due to differences in risk-taking behavior across the age spectrum, as well as representing a population with less comorbidities which also contributes to PDD risk. Among 14 studies that examined the role of stimulants, 12 found that stimulant use in combination with OUD had higher rates of PDD - including one prospective cohort study with intervention. This is not surprising as patients using stimulants may experience uncontrolled cravings from lack of access to methamphetamine in the hospital setting, even if MOUD is provided. Multiple studies found PDD occurred more frequently in patients with socioeconomic challenges including lack of insurance, housing instability, and poverty, further emphasizing the vulnerability of this patient group.

In terms of possible protective factors, 3 studies found that patients with more severe medical comorbidity score had lower incidence of PDD; this is expected as patients must be well enough to physically leave a hospital. Those patients with additional comorbid conditions, who are presumably sicker may desire the treatment of their underlying comorbidities and be more willing to stay in the hospital for treatment.

One central challenge in understanding patient-level risk, is that protocolized assessment of severity of opioid use disorder, a driver of withdrawal and ultimately PDD, is quite limited. For instance, 2 patients may meet the DSM-V criteria for “opioid use disorder, severe”—one person using intranasal fentanyl daily and someone who is injecting 20-30 pills of fentanyl daily—however, these 2 individuals are not likely at the same risk of PDD related to opioid withdrawal.

### Clinical Care Factors

Twelve studies explored the association between MOUD treatment in the hospitalized setting and PDD and reached differing conclusions.^28^^,^^30^^,^^33^^,^^35^^,^^36^^,^^55^^,^^57^^,^^64^^,^^70^, ^71^, ^72^, ^73^ Six authors found that there was decreased PDD with MOUD,^28^^,^^36^^,^^55^^,^^64^^,^^71^^,^^72^ 5 found similar incidence of PDD^30^^,^^33^^,^^35^^,^^57^^,^^70^ and one author reports a higher incidence of PDD with patients receiving MOUD.^73^ Of these MOUD studies, one study utilized matched controls^33^ and found similar incidence, while all other studies utilized non-randomized, retrospective samples. One RCT found a higher rate of PDD among patients treated with long-acting injectable buprenorphine compared to treatment as usual.^81^

Much of the literature treats MOUD as a binary exposure (eg, either received any MOUD or none), which may not capture how clinical context may affect patient experience and ultimately PDD. Authors assessed PDD by chart review as well as discharge disposition codes for large administrative data sets. While chart review is the most accurate way to assess whether a discharge was planned, large datasets (eg, *National Inpatient Sample*) provide sufficiently detailed discharge variables such that the different means of adjudication would not explain differences in findings between these approaches. There is a lack of large, high-quality studies on PDD and MOUD: only 2 studies of MOUD include national samples^35^^,^^55^ with the rest of the literature drawn from small, retrospective cohort studies drawing from single health systems.

Three recent studies explore the MOUD and PDD relationship in more detail.^55^^,^^57^^,^^72^ Alrawashdeh et al. performed a retrospective cohort study using datasets from 362 US hospitals between 2009 and 2015 that examined withdrawal management strategies among 127,158 hospital encounters. This study is unique in that it analyzed MOUD exposure as a temporal variable during the first 2 days of hospitalization (a time when PDD rates are the highest) and found lower incidence of PDD among MOUD treated patients.^55^ Two other studies evaluated timing and dosage of MOUD at this granular scale. One study of patients in San Francisco found high rates of MOUD (60.6%) of which 92.8% received methadone—in this sample patients with methadone initiation did not have lower incidence of PDD but patients who on methadone maintenance at admission had lower incidence of PDD.^57^ Another study that analyzed PDD in the context of MOUD dose changes over time among 928 patients hospitalized within the University of Pennsylvania Health System in 2016 demonstrated a decreased incidence of PDD among patients treated with any opioid vs no opioid and that patients with dose reductions in methadone had higher incidence of PDD.^72^

The conflicting findings between MOUD and PDD may be explained by differences in MOUD selection, timing, dosage, baseline opioid tolerance, prior fentanyl exposure, uncontrolled cravings (methamphetamines), withdrawal from other substances (xylazine, medetomidine) as well as other variables such as stigma—all of which would be expected to contribute to patient experience, withdrawal and potential PDD. Additionally, several authors have asserted that MOUD treatment correlates with the severity of OUD—such that patients who receive MOUD represent a population at higher risk of withdrawal and PDD.^57^^,^^73^ A corollary finding is that in-hospital use of illicit opioids is a risk factor of PDD.^30^ These results suggest that MOUD dosage and timing are mediators of the effect between treatment with MOUD and PDD but that this pathway is context-specific.

Other promising clinical interventions regarding MOUD and early withdrawal management include rapid methadone titration and use of short-acting full agonist opioids to more quickly treat withdrawal. In retrospective case series utilizing rapid methadone titration protocols (ex. giving daily doses up to 60mg-80mg-100mg on days 1-3), PDD rates were only 4%-5%. However, these studies generally have small sample sizes and lack comparator groups.^46^^,^^76^, ^77^, ^78^ Similarly, a case series on pilot of sOAT protocol by Thakrar et al. found that patients treated with mean opioid doses 200-320 MME during the first 24 hours experienced a PDD rate of 43.5% compared to 69% in a historical comparator group.^79^ This finding can be compared to other small case series, not included in this review, which have sought to explore the utility of high-dose sOAT for initial management of patients with exposure to fentanyl.^41^^,^^88^^,^^89^ Further research is needed to determine impact of different OUD treatment strategies on rates of PDD.

### Addiction Consultation Services

The impact of ACS on PDD is mixed. Studies by Buresh et al.^90^ and Marks et al. reported that patients who received consultations had a decreased incidence of PDD^39^ however that finding was not replicated in other studies.^33^^,^^82^^,^^83^ It is striking that in Marks et al. there was almost no MOUD in the treatment-as-usual (TAU) group (17.2%) compared to the group that received a consult (86.8%). This study was also completed in 2016-2018 which may reflect lower rates of MOUD used in hospital systems. Additionally, causal interpretation of these studies is limited due to use of non-randomized, retrospectively collected data. Karol et al. used a quasi-randomized design to assign patients for an alert for proactive addiction consultation but did not report baseline characteristics between the groups that received addiction consult; there was a non-significantly lower incidence of PDD in the addiction consult group (7.5% vs. 10.2% *p* = .619).^82^ Of the other studies of ACS, one study used matched historical controls (drawn from 2019 compared to 2021 after the ACS launched)^33^, 2 utilized convenience sampling,^39^^,^^83^ and one used ICD-10 codes for history of OUD (Buresh et al.). These methodological issues are significant as patients who receive addiction consults may have different baseline characteristics and be characterized by significant confounding by indication. This is represented in the recent study by Buresh et al. study, rate of PDD was higher among patients with any SUD seen by ACS (any SUD) in unadjusted analysis (OR 1.21, CI 1.06-1.38), however the association reversed in adjusted analysis (aOR 0.81, 95% CI 0.71-0.93). Indeed, a recent systematic review of the effects of ACS on care for patients with OUD found 27% of studies observed positive impact compared to control, 15% found no difference and 58% did not cf against a control, highlighting the limitations of the existing literature.^29^^,^^90^ Beyond the effects of ACS on PDD, these studies often report outcomes such as increased patient engagement, increased provider comfort with OUD treatment, improved patient satisfaction, increased antibiotic treatment completion rates, and improved transitions to outpatient care.^39^^,^^86^^,^^91^, ^92^, ^93^, ^94^

### Rapid Methadone Titration, Novel Buprenorphine Initiation Strategies and Short-Acting Opioid Agonists

With the growing recognition that patients with exposure to fentanyl will have higher tolerance for opioid medications, multiple authors have explored rapid methadone titration (RMT) protocols and associated PDD.^46^^,^^76^, ^77^, ^78^^,^^95^ Instead of the conventional approach to methadone initiation with 30-40mg on the first day with gradual increases, RMT protocols reach higher doses of methadone in a shorter period. RMT protocols conducted at single centers with ACS have shown promise in managing withdrawal symptoms efficiently and are characterized by low rates of PDD (4%-5%) and infrequent adverse events. However, these studies generally have small sample sizes and lack comparator groups.^46^^,^^76^, ^77^, ^78^

Alternatively, PDD has been reported with the use of low-dose buprenorphine induction (LDBI)^96^, ^97^, ^98^, ^99^ as well as injectable buprenorphine.^81^^,^^100^ Similarly, high dose or macro dose treatment with buprenorphine, generally thought of as dose of buprenorphine >12 mg of SL-buprenorphine, have been demonstrated to have high levels of tolerability and low rates of precipitated withdrawal but their impact on PDD has not been studied explicitly.^101^

One case series included in this review reports decreased PDD for patients treated with short-acting opioid agonists in addition to MOUD.^79^ In this study, patients treated with mean opioid doses 200-320 MME during the first 24 hours experienced a PDD rate of 43.5% compared to 69% in a historical comparator group.^79^ This finding can be compared to other small case series, not included in this review, which have sought to explore the utility of high-dose opioid agonist therapy for initial management of patients with exposure to fentanyl.^41^^,^^80^^,^^88^^,^^89^ These findings support a dose-response relationship between PDD and treatment of OUD, however, must be interpreted with caution and are preliminary. This work agrees with qualitative research on the individual drivers of PDD which have consistently found that patients with OUD cite undertreated pain, withdrawal, stigma, and social needs outside the hospital as contributing to PDD.^102^, ^103^, ^104^, ^105^, ^106^, ^107^

### Other Clinical Interventions

There have been limited reports on the effect of other clinical interventions.^108^, ^109^, ^110^, ^111^, ^112^, ^113^, ^114^ Two studies reported less PDD in patients with OUD who underwent surgical intervention.^5^^,^^54^ This may be related to the severity of comorbid illness with patients with infective endocarditis requiring cardiac surgery having severe preoperative heart failure and also being less able to leave the hospital.

Studies of specialized teams have reached different conclusions for PDD. These interdisciplinary teams typically consist of physicians trained in addiction medicine, clinical pharmacists, social workers, case managers, peer recovery coaches, and in some centers, are integrated with infectious disease physicians as well.^115^ One prospective cohort study using controls reported an intervention including a group of specialists including addiction medicine, infectious diseases, pain stewardship pharmacy and peer counselor and social workers called the severe injection-related infection (SIRI) team showed remarkable reduction in PDD compared to historical controls (17% vs. 37.1%)^86^ In contrast, another group reported null findings of specialized team approach using a retrospective, pre-post design. In this study, collaborative care by a team made up of cardiac surgeons, infectious diseases, addiction medicine, cardiology as well as a broader group that included interventional cardiologists, neurologists, neurosurgeons, nurses, pharmacists, peer recovery coaches, case managers and social workers did not see a drop in PDD (11.1% vs. 5.3%).^84^ These results may be explained if the latter team was more focused on operationalizing surgical management rather than focused on patient-centered needs such as withdrawal and pain control. More research is needed on these multidisciplinary approaches.

### Systems-Level Factors

There were only 2 manuscripts to study systems level factors in relation to PDD among hospitalized patients with OUD.^54^^,^^58^ Both utilized retrospective, observational cohort designs. The findings that larger hospitals have decreased risk of PDD may be related to staffing and experience; however, there are common systems level issues across sizes. Hospitals have been described as a “risk environment” for patients with substance use disorder: instead of providing comfort and promoting health the inpatient setting may be a source of trauma and pose a threat to health due to an underlying lack of trust and understanding between care teams and patients.^116^^,^^117^ Similarly, medical providers have described feeling unable to adequately address pain and withdrawal, trapped between restrictive hospital policy and patient wishes.^91^^,^^118^^,^^119^ Hospitals present restrictive environments for patients—often limiting visitors, restricting smoking, controlling the ability to leave the floor, and invading privacy.^120^^,^^121^ Additionally, daily hospital routines such as phlebotomy are a source of friction with the care team as patients with OUD may have decreased venous access and have unpleasant experiences with repeated attempts at blood draws. It is conceivable that systems level features, with certain hospitals being more or less responsive to patient-centered care for OUD, contribute to PDD. This is echoed in the qualitative literature as well: patients with OUD also report that more intangible qualities of care such as empathy and being made to feel welcome constitute an important part of decision to remain hospitalized.^122^, ^123^, ^124^ Patients with history of trauma or incarceration can feel particularly vulnerable when separated from friends and family, face interruptions to sleep, and a lack of privacy. Efforts to improve patient’s sense of autonomy and make hospital care less invasive may lead to improved experience for patients and less desire to leave the hospital setting. Larger hospitals and academic medical centers often have more resources for addiction care than smaller and rural facilities. There are certainly geographic differences in access to methadone across rural and metropolitan areas which affect initiation of this medication as a long-term option for patients who are hospitalized. Misunderstanding of federal regulations regarding methadone lead many hospitals to misinterpret 3-day rule and not initiate methadone, as well as applying outpatient dose restrictions to inpatient settings. Additionally, systematic structural issues such as lack of insurance and variability in access to ongoing care can limit linkage to ongoing MOUD.

**Figure 2:**
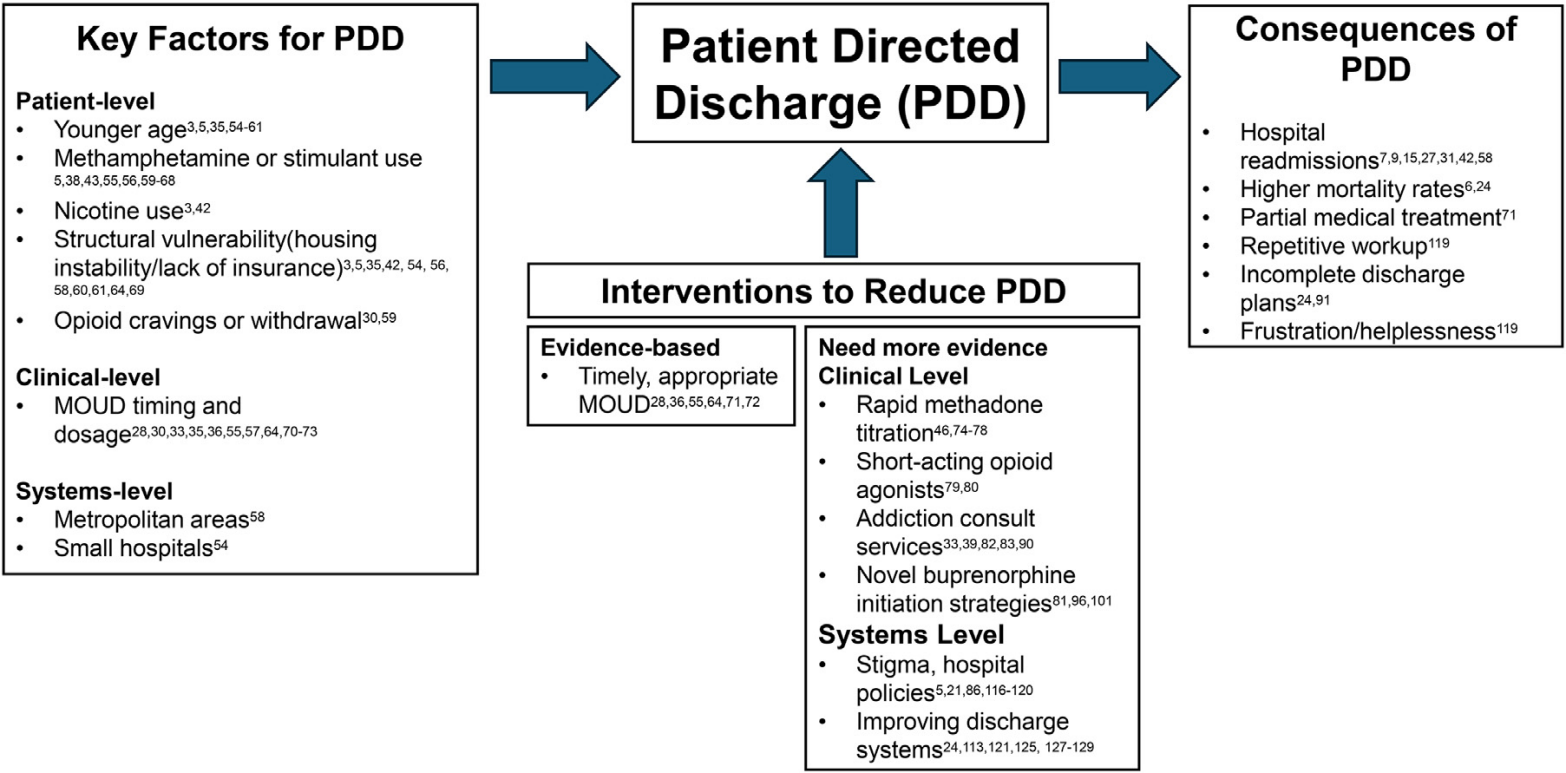
Patient directed discharge among patients with opioid use disorder: risk factors, interventions, and consequences.

Another aspect of systems-level care limitations possibly driving PDD is prolonged hospitalizations related to long-term antibiotics.^125^ Despite OUD being a protected disability, placement in post-acute care facilities, such as skilled nursing facilities (SNF) is often difficult for this population.^126^ This is largely driven by stigma towards patients with substance use disorders, and that SNFs are often not equipped with staffing to support patients with SUD.^127^^,^^128^ Many homecare companies do not offer to provide outpatient intravenous antibiotic treatment for persons with history of intravenous drug use due to concerns about liability from patient misuse which result in extended hospitalizations to complete antibiotics, ultimately putting patients at ongoing risk of PDD.^129^ Factors affecting patient-directed discharge are summarized in Figure 2.

## Conclusion

PDD is a frequent event among hospitalized patients with OUD which is associated with risk factors at the patient, clinical care and systems level. There is emerging evidence that clinical interventions may be linked to decreased rates of PDD: the use of MOUD, ACS engagement, the use of full opioid agonists, rapid methadone titration protocols and flexible buprenorphine initiations represent areas of emerging evidence. Additionally, work is underway to transform hospitals into more welcoming spaces for patients through staff training and improving policies to reduce stigma, and in-hospital harm reduction interventions. These efforts are exciting and will require further evaluation in the period of fentanyl-predominant opioid supply to determine effectiveness and feasibility in improving care and preventing PDD.

Our goal in this review is to illustrate the state of the literature regarding OUD and PDD and highlight where evidence has accumulated supporting certain approaches. Overall, there is limited high-quality evidence to guide current practice; particularly prospective data, analysis at the systems level, and longitudinal metrics. Future research should prioritize development of clinically relevant metrics including timing and dosing of MOUD, evidence from longitudinal cohorts, and more rigorous evaluation of randomized interventions to better illustrate how to best serve high-risk patients hospitalized with OUD and reduce PDD.

## CRediT authorship contribution statement

**William M. Garneau:** Writing – review & editing, Writing – original draft, Validation, Software, Resources, Methodology, Investigation, Formal analysis, Data curation, Conceptualization. **Severyn Kushmeliuk:** Writing – review & editing, Writing – original draft, Methodology, Investigation, Data curation. **Dustin P. Kee:** Writing – review & editing, Writing – original draft, Validation, Methodology, Investigation. **Kelly A. Gebo:** Writing – review & editing, Writing – original draft, Supervision, Project administration, Investigation, Formal analysis. **Megan E. Buresh:** Writing – review & editing, Writing – original draft, Validation, Supervision, Resources, Project administration, Methodology, Investigation, Data curation, Conceptualization.

## Declaration of competing interest

KAG receives royalties from UpToDate, non-paid position at Pfizer, and personal consulting from Spark HealthCare, Premier HealthCare, Harrison Consulting and MedEd Learning. WMG reports serving as a scientific advisor to Gilead Sciences, Inc and Premier, Inc; and owning stock in Abbott Laboratories, Astrazeneca Pharmaceuticals LP, Danaher, UnitedHealth Group, Inc., IQVIA, Stryker Corporation, Eli Lilly and Company, and Intuitive Surgical Inc.

## Appendix Supplementary materials

Image, application 1
