# Supplementary material for: Patient-Directed Discharge Among Hospitalized Persons With Opioid Use Disorder in the Fentanyl Era: A Scoping Review
Source: Am J Med Open. 2026 Feb 28;15:100130. doi: 10.1016/j.ajmo.2026.100130 (PMC13089212; doi:10.1016/j.ajmo.2026.100130)
Supplement: Supplementary file 1 [file mmc1.docx]

**Appendix**

Search terms used for various databases

**CINAHL**

(XB “fentanyl” or MH “fentanyl” OR XB “heroin” OR MH “heroin” OR XB “opioid epidemic” OR MH “opioid epidemic” OR XB “methadone” OR MH “methadone” OR XB “buprenorphine” OR MH “buprenorphine” OR XB “substance abuse” OR MH “substance abuse” OR XB “substance dependence” OR MH “substance dependence” OR XB “persons with substance use disorders” OR MH “persons with substance use disorders” OR MH “substance use disorders” OR XB “substance use disorders” OR MH “buprenorphine” OR XB “buprenorphine” OR XB “buprenorphine, naloxone drug combination” OR XB “suboxone” OR XB “Opioid-Related Disorders” OR XB “opioid-related disorder” OR XB “opioid use disorder” OR “opioid use disorders” OR XB “opiate addiction” OR XB “medication for opioid use disorder” OR XB “MOUD” OR XB “heroin dependence”) AND (MH " Treatment Refusal" OR XB "Treatment Refusal " OR XB “refusal of care” OR XB “patient refusal of treatment” OR XB “Healthcare Avoidance” OR XB “against medical advice” OR XB “leaving against medical advice” OR XB “patient-directed discharge” OR XB "patient refusal of treatment")

**PUBMED**

("2015/01/01"[Date - Publication] : "3000"[Date - Publication])AND (("Treatment Refusal"[MeSH Terms] OR "treatment refusal*"[Title/Abstract] OR "Healthcare Avoidance"[Title/Abstract] OR "Refusal of care"[Title/Abstract] OR "patient refusal of treatment*"[Title/Abstract] OR "Leaving Against Medical Advice"[Title/Abstract] OR "Leave Against Medical Advice"[Title/Abstract]) OR ("Patient-directed discharge"[Title/Abstract])) AND (“fentanyl”[MeSH Terms] OR “fentanyl” [Title/Abstract] OR "Methadone"[MeSH Terms] OR "buprenorphine, naloxone drug combination"[MeSH Terms] OR "Methadone"[Title/Abstract] OR "suboxone"[Title/Abstract] OR "Opioid-Related Disorders"[MeSH Terms] OR "Opioid-Related Disorders"[Title/Abstract] OR "Opioid-Related Disorder"[Title/Abstract] OR "opioid use disorder"[Title/Abstract] OR "opioid use disorders"[Title/Abstract] OR "opiate addiction"[Title/Abstract] OR "medication for opioid use disorder"[Title/Abstract] OR "MOUD"[Title/Abstract] OR "heroin"[ Title/Abstract] OR "heroin dependence" [Title/Abstract] OR "heroin"[MeSH Terms] or "heroin dependence" [MeSH Terms])

**Embase**

('treatment refusal'/exp OR 'treatment refusal*':ti,ab OR 'healthcare avoidance':ti,ab OR 'refusal of care':ti,ab OR 'patient refusal of treatment*':ti,ab OR 'leaving against medical advice':ti,ab OR 'leave against medical advice':ti,ab OR 'patient-directed discharge':ti,ab) AND ('fentanyl'/exp OR 'fentanyl':ti,ab OR 'methadone'/exp OR 'buprenorphine plus naloxone'/exp OR 'methadone':ti,ab OR 'suboxone':ti,ab OR 'opioid-related disorder'/exp OR 'opioid-related disorders':ti,ab OR 'opioid-related disorder':ti,ab OR 'opioid use disorder':ti,ab OR 'opioid use disorders':ti,ab OR 'opiate addiction':ti,ab OR 'medication for opioid use disorder':ti,ab OR 'moud':ti,ab OR 'heroin':ti,ab OR 'heroin dependence':ti,ab OR 'diamorphine'/exp OR 'heroin dependence'/exp) AND (2015:py OR 2016:py OR 2017:py OR 2018:py OR 2019:py OR 2020:py OR 2021:py OR 2022:py OR 2023:py OR 2024:py OR 2025:py) AND 'article'/it AND [english]/lim

**Cochrane**

([mh "Treatment Refusal"] OR ("treatment" NEXT refusal*):ti,ab OR "Healthcare Avoidance":ti,ab OR "Refusal of care":ti,ab OR ("patient refusal of" NEXT treatment*):ti,ab OR "Leaving Against Medical Advice":ti,ab OR "Leave Against Medical Advice":ti,ab OR "Patient-directed discharge":ti,ab) AND ([mh fentanyl] OR fentanyl:ti,ab OR [mh Methadone] OR [mh "buprenorphine, naloxone drug combination"] OR Methadone:ti,ab OR suboxone:ti,ab OR [mh "Opioid-Related Disorders"] OR "Opioid-Related Disorders":ti,ab OR "Opioid-Related Disorder":ti,ab OR "opioid use disorder":ti,ab OR "opioid use disorders":ti,ab OR "opiate addiction":ti,ab OR "medication for opioid use disorder":ti,ab OR MOUD:ti,ab OR heroin:ti,ab OR "heroin dependence":ti,ab OR [mh heroin] OR [mh "heroin dependence"])
